# In vivo CAR-cell therapy: current challenges and emerging therapeutic advances

**DOI:** 10.1186/s43556-026-00447-y

**Published:** 2026-04-20

**Authors:** Yi-Min Yang, Bo Bao, Yu-Hao Cao, Jin Yao, Yu-Fan Ding, Yi-Yang Hu, Fan Fan, Jun-Long Zhao

**Affiliations:** 1 State Key Laboratory of Holistic Integrative Management of Gastrointestinal Cancers, Department of Medical Genetics and Developmental Biology, Fourth Military Medical University, Chang-Le Xi Street #169, Xi’an, 710032 China; 2 Basic Medical College, Fourth Military Medical University, Xi’an, 710032 China

**Keywords:** In vivo CAR therapy, Synthetic biology, Gene editing, Delivery system, Artificial intelligence

## Abstract

In vivo chimeric antigen receptor (CAR) cell therapy is undergoing a transformative shift from conventional ex vivo manufacturing toward in situ cellular editing, aiming to generate functional CAR-engineered immune cells directly within patients through targeted vector delivery, thereby significantly enhancing therapeutic accessibility and applicability. While rapid advances have been made in both viral (lentiviral and adeno-associated viral vectors) and non-viral (lipid nanoparticle) delivery platforms, along with the expansion of effector cell lineages including CAR-T, CAR-NK, and CAR-M, critical translational bottlenecks remain. These include insufficient delivery precision, limited cellular persistence, immunosuppressive tumor microenvironment (TME) resistance, and challenges in safety controllability. This review systematically examines the working mechanisms and limitations of current delivery platforms for in vivo gene transfer. It provides a comprehensive comparison of how CAR-T, CAR-NK, and CAR-M platforms employ distinct yet complementary strategies to address tumor heterogeneity, solid tumor physical and immune barriers, and the specificity constraints of in situ editing. Furthermore, we highlight emerging frontiers such as artificial intelligence-guided personalized therapy design, smart delivery systems (logic-gated CARs, circular RNA vectors), and the development of multicellular synergistic “synthetic immune systems.” By integrating multidisciplinary perspectives, this review not only offers a comprehensive roadmap bridging fundamental mechanisms to clinical translation but also lays a theoretical and technical foundation for advancing the next generation of safe, precise, and efficacious in vivo CAR therapies.

## Introduction

Genetically engineered immune-cell therapy, particularly CAR-T cells, has revolutionized the treatment of hematologic malignancies and ushered in a new era of cancer immunotherapy [1, 2]. Central to this success is the ex vivo paradigm [3], in which a patient’s or donor’s immune cells are genetically engineered, expanded, and reinfused to exert therapeutic effects. However, inherent limitations of this model—including complex manufacturing, lengthy timelines, high costs, and dependence on patient-specific cell quality—severely constrain its broad application and accessibility [4–7].

To overcome these barriers, the immunotherapy field is undergoing a profound shift from”cell as a drug” toward”the patient’s body as a bioreactor” [8–12]. This has given rise to in vivo CAR-cell therapy, whose core objective is to directly reprogram endogenous immune cells (T cells, NK cells, macrophages) in situ through the targeted in vivo delivery of genetic vectors, conferring transient or durable tumor-targeting capabilities [13, 14]. By simplifying complex cellular manufacturing into one or more precise in vivo administrations, this strategy holds the potential to streamline processes, reduce costs, and expand therapeutic indications to solid tumors, autoimmune disorders, and even infectious diseases [15].

Yet this promising vision introduces unprecedented scientific and translational challenges. First, delivery platforms form the cornerstone of accurate, efficient, and safe in situ editing [16, 17]. Conventional viral vectors (lentivirus, adeno-associated virus) and non-viral systems (lipid nanoparticles, polymeric carriers) each present distinct trade-offs in transduction efficiency, persistence, immunogenicity, and targeting specificity [18, 19]. A primary challenge is to tailor these platforms to the biological properties of different immune-cell types (T, NK, and macrophage lineages) to achieve effective in vivo programming [20]. Second, CAR cells generated in vivo face formidable survival and functional hurdles: they lack exogenous cytokine support, are constrained by the host’s pathological state and the immunosuppressive tumor microenvironment, and are prone to functional exhaustion. As a result, their expansion capacity and durability typically fall short of those achieved with ex vivo-produced counterparts [21, 22]. Moreover, safety risks are more complex, encompassing “on-target, off-tumor” toxicity due to tumor heterogeneity and antigen promiscuity, cytokine release syndrome (CRS) from unpredictable in vivo expansion, and long-term risks associated with viral-vector insertional mutagenesis or off-target gene editing [23, 24].

Currently, the field stands at a critical juncture between proof-of-concept and clinical translation. Early-stage trials have begun to validate the feasibility of various approaches while also revealing core bottlenecks such as inconsistent delivery efficiency and limited cellular persistence [25, 26]. Future breakthroughs will hinge on deeper interdisciplinary integration and intelligent technological advancement. Key directions include: developing next-generation smart delivery systems (logic-gated, microenvironment-responsive carriers) [27, 28]; constructing multicellular synergistic “synthetic immune systems” (CAR-T/CAR-NK/CAR-M) to overcome solid-tumor barriers [29, 30]; and leveraging biomarkers and artificial intelligence to enable precise patient stratification and personalized therapeutic design [9, 31, 32].

This review aims to systematically outline the latest progress, core challenges, and future directions in in vivo CAR-cell therapy. We will first analyze and compare the technical principles and application landscapes of major delivery platforms (viral and non-viral). Next, we will examine the key biological barriers—including cell survival, exhaustion, and microenvironmental suppression—that limit in vivo CAR-cell function and discuss emerging strategies to address them. We then summarize the current status and insights from early clinical translation efforts. Finally, we look ahead to pathways for intelligent therapy evolution through personalized stratification, AI-assisted design, and cross-disciplinary innovation. Through this comprehensive discussion, we hope to present a coherent picture and a forward-looking roadmap for in vivo CAR-cell therapy as it advances from technological exploration toward clinical reality.

## Delivery system: serving as a vector for mediating gene transduction in vivo

### Viral vectors: the cornerstone of stable expression

The core of CAR immune cell therapy lies in using genetic engineering to efficiently and stably deliver artificial gene sequences encoding CARs into effector cells. This process endows the cells with a new capacity to target and eliminate tumors [33]. To achieve this goal—particularly to ensure durable CAR molecule expression for long-term efficacy—gene-integrating viral vectors, notably Lentiviral Vectors (LVs) and γ‑retroviral vectors, have become the undisputed cornerstone of current CAR cell manufacturing [34, 35]. These two vector platforms serve as the production standard for nearly all approved CAR‑T products (Yescarta) and for the vast majority of CAR cell therapies entering clinical studies [36–38].

#### Retrovirus

As a pioneering integrated vector platform, γ‑retrovirus has opened the door to gene therapy through its unique biological mechanism [34]. When viral particles fuse with the target cell membrane, the double‑stranded DNA produced by reverse transcription of the single‑stranded RNA genome in the cytoplasm enters the nucleus during mitosis—specifically when the nuclear envelope breaks down—and is stably integrated into the host genome via viral integrase [39]. This gene‑integration capability enables durable CAR molecule expression, allowing genetically engineered immune cells to propagate the transgene stably through cell division [40].

Clinically, retroviral vectors have enabled the critical leap from bench to bedside. Kochenderfer et al. [41] first used this platform to generate anti‑CD19 CAR‑T cells and achieved complete remission in patients with B‑cell lymphoma, marking the first demonstration that genetically reprogrammed T cells could eliminate malignant tumors in vivo. Subsequently, Savoldo et al. [42] demonstrated through comparative clinical trials that retroviral vector‑mediated co‑expression of the CD28 costimulatory domain significantly enhanced CAR‑T cell persistence and expansion in patients, thereby defining the core architecture of modern second‑generation CAR designs.

However, for in vivo transduction scenarios, Poorebrahim et al. [43] highlight a major biological limitation: retroviral integration complexes cannot cross an intact nuclear membrane and must rely on host‑cell mitosis (with nuclear envelope breakdown) to access the genome. Consequently, resting T cells—which are largely quiescent in vivo—are poorly transduced. Experimental data show that transduction efficiency in resting T cells is typically below 5%, far lower than the 50%–80% achievable in activated T cells. This bottleneck significantly limits the efficiency of direct in vivo reprogramming and has driven a shift in research focus toward lentiviral vectors.

#### Lentivirus

Unlike retroviruses, lentiviral vectors are engineered for active nuclear import, enabling them to efficiently infect non-dividing cells [44]. When vector particles interact with target cells, they deliver the RNA genome containing the CAR gene into the cytoplasm. Following reverse transcription into DNA, the viral integrase mediates integration into the host genome in a random or semi-random manner [45]. This integrated feature ensures the stable transmission of CAR molecules during cell division, providing long-term and reliable “arming” for a variety of effector cells such as CAR-T, CAR-NK, and CAR-M [46].

With technological evolution, delivery platforms have advanced from ex vivo engineering toward in vivo engineering [47]. Central to this transition is the development of viral vectors capable of selectively targeting specific immune-cell subsets. In exploring T-lymphocyte-specific transduction, Pfeiffer et al. [48] first demonstrated the feasibility using a CD8-targeted LV (CD8-LV) in a humanized mouse model. Subsequently, Frank et al. [49] and Michels et al. [50] achieved an in vivo transduction rate of up to 40% by conjugating anti-CD3 antibody fragments to the viral surface, further confirming the high efficiency of surface-engineering strategies for precisely activating and transducing resting T cells.

Concurrently, this in vivo engineering paradigm is extending into the innate immune spectrum. Building on the enhancement of lentiviral transduction efficiency in NK cells by Hayal et al. [51] and the validation of macrophage-targeted modification by Klichinsky et al. [52], the direct in situ generation of CAR-NK and CAR-M cells—which possess enhanced solid-tumor infiltration capacity—is emerging as a key research focus following advances in CAR-T technology.

#### Adeno-associated Virus (AAV)

Among non-integrating vectors, AAV has emerged as a prominent candidate for in vivo gene delivery owing to its low immunogenicity and favorable safety profile [53]. Unlike integrating vectors such as retroviruses or lentiviruses, the AAV genome remains predominantly episomal after nuclear entry, with random integration occurring only at very low frequencies [54]. This non-integrating nature enables transgene expression without altering the host genome, thereby substantially reducing the risk of insertional mutagenesis. However, AAV faces intrinsic biological barriers for in situ T-cell transduction. First, T cells lack high-affinity receptors for common AAV serotypes (e.g., heparin sulfate proteoglycan) [55]. Second, as an exogenous single-stranded DNA virus, AAV readily triggers intrinsic antiviral defenses in T cells, leading to pre-transcriptional genome degradation and resulting in initially very low transduction efficiencies, often below 1% [56, 57].

Despite these hurdles, capsid engineering has led to notable progress. Nawaz et al. [58] achieved an in vivo CAR gene transduction efficiency of approximately 10%—a significant increase from the < 1% typically seen with conventional serotypes—by systematically optimizing serotype selection and delivery via a single intravenous infusion of the highly infectious AAV-DJ variant. This study highlighted the clinical potential of AAV for in situ generation of functional CAR-T cells.

The suitability of these three vector platforms for in vivo editing varies considerably. Retroviral vectors are strictly dependent on cell division, which limits their ability to transduce resting lymphocytes and constrains their utility for in vivo therapies [37, 47]. In contrast, lentiviral vectors—capable of efficiently transducing non-dividing cells and amenable to surface engineering—have been established by Hunter et al. as the leading platform for durable in vivo reprogramming [59]. Although AAV has a limited cargo capacity, its non-integrating feature and low immunogenicity position it as a safe alternative that avoids insertional mutagenesis risks [36, 38].

### Non-viral vector systems: the frontier of security and flexibility

**Fig. 1:**
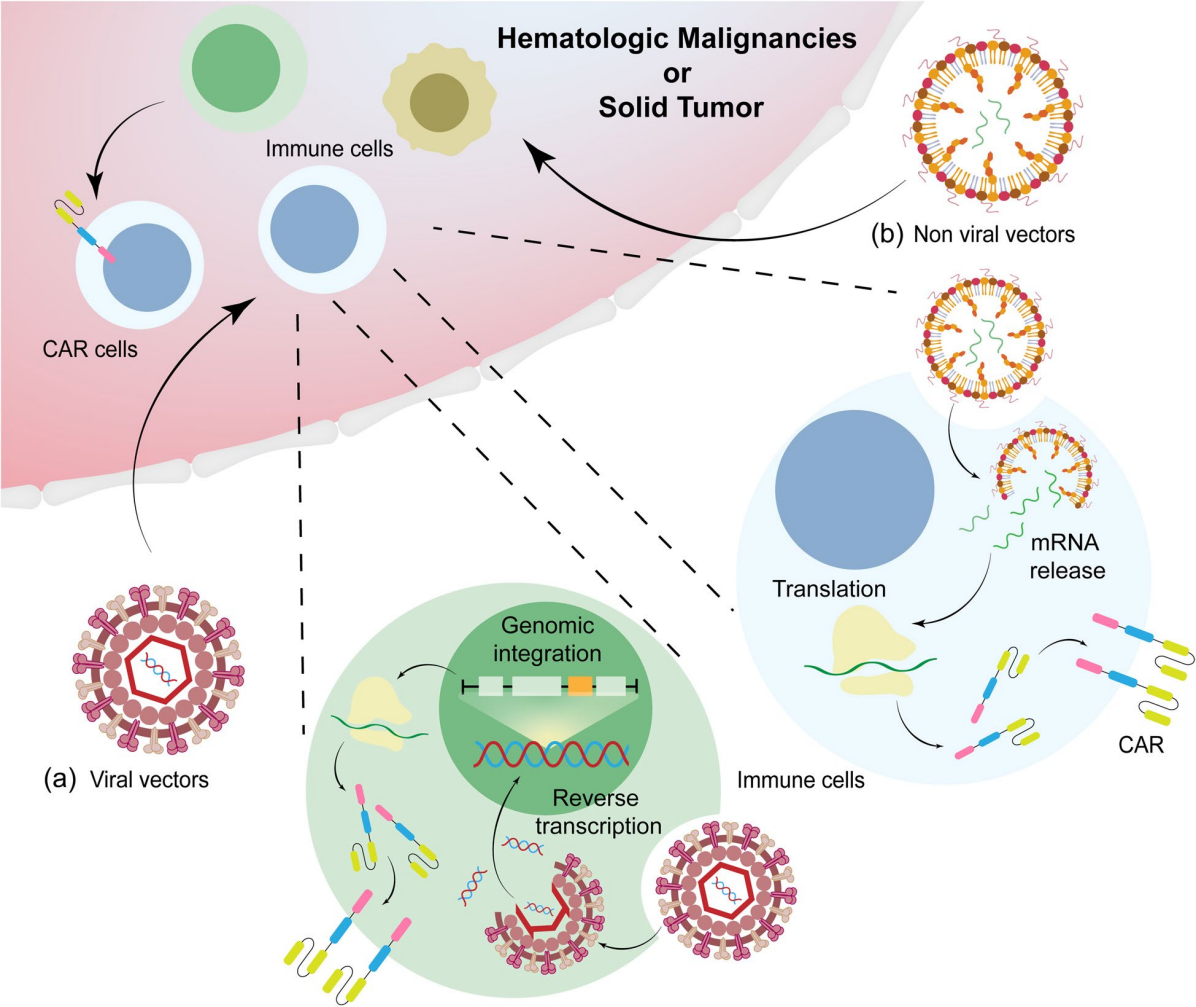
The in vivo CAR cell therapy mechanism of viral/non-viral vectors. This figure illustrates the two principal gene delivery strategies employed in the production of in vivo CAR cell therapies. The cell engineering workflow bifurcates into two distinct pathways: **a** The viral vector approach: lentiviral or retroviral vectors are used to deliver the CAR transgene into the host cell nucleus. Following reverse transcription, the genetic material undergoes genomic integration, enabling long-term and stable expression of the CAR protein. **b** The non-viral vector approach: In vitro-transcribed mRNA serves as the genetic payload. It is directly introduced into the cytoplasm via physical methods such as electroporation. The mRNA is then translated to produce the CAR protein. This method avoids genomic integration, resulting in transient but high-level expression of the CAR construct, after which the mRNA is naturally degraded within the cell

Although viral vectors have established a “gold standard” in ex vivo CAR‑cell manufacturing, their inherent immunogenicity, limited cargo capacity, and the unavoidable risk of insertional mutagenesis pose significant translational barriers when applied directly to in vivo gene editing [38, 60]. In this context, non‑viral vector systems—particularly lipid nanoparticles (LNPs), polymeric nanoparticles, and inorganic nanoparticles—are reshaping the landscape of immune‑cell gene therapy, owing to their favorable biosafety profiles, low immunogenicity, and scalability for industrial production [36, 61] (Fig. 1).

#### In vivo T cell reprogramming

In the field of in vivo reprogramming of T cells, LNPs have emerged as the most clinically advanced non-viral delivery platform. To address the natural barrier of inefficient transduction in resting T cells, research has focused on optimizing lipid chemical structures to enhance cell-type specificity and uptake. Billingsley et al. [62] constructed a proprietary ionizable lipid library through high-throughput screening and tuned the pKa of LNPs to develop formulations with extrahepatic tropism. This optimization enhanced mRNA expression in splenic and lymphoid tissues by more than tenfold compared to conventional MC3-based LNPs, thereby enabling efficient in vivo T-cell transduction.

Furthermore, to simplify the complex antibody-conjugation processes and adapt to the heterogeneity of the in vivo environment, biomimetic design strategies have been introduced. Zhang et al. [18] developed a cardiolipin-based LNP that leverages endogenous cellular mechanisms for T-cell transfection—without requiring antibody modification. This system achieved approximately fivefold higher transduction efficiency in splenic T cells compared to conventional lipid formulations and significantly improved the robustness of in vivo gene delivery.

#### Modification of macrophages

In addition to T cells, non-viral vectors also show a high degree of cell type-specific adaptation in the modification of innate immune cells. For macrophages (CAR-M), taking advantage of their natural strong phagocytic ability is a key strategy to improve the delivery efficiency, while also taking into account the immune remodeling of the TME. Han et al. [63] designed a co-delivery strategy to use LNP to package CAR mRNA and immune agonist simultaneously, which not only transfected macrophages efficiently, but also significantly increased the proportion of M1 tumor suppressor phenotype in the tumor microenvironment from less than 20% to more than 75%, and simultaneously realized immune remodeling. A similar strategy has also been applied to non-tumor diseases. Du et al. [64] used LNP to reprogram macrophages to treat myocardial ischemia–reperfusion injury and significantly reduced the Infarct Size of the injured mice. However, the study by Argueta et al. [65] further confirmed that LNP optimized by specific lipid formulation could specifically deliver CAR gene to myeloid cells and achieve tumor regression in solid tumor model, which overcomes the problem of low transduction efficiency of traditional carriers to myeloid cells.

#### Delivery of NK cells

In contrast, NK cells are naturally resistant to lipofection and are susceptible to cytotoxicity, prompting a shift toward non-lipid materials for vector development. Polymeric systems offer an ideal alternative due to their structural controllability and tunable charge density. Gharatape et al. [66] demonstrated that cationic polymers based on poly β-amino esters (PBAEs) can form stable nanocomplexes via electrostatic condensation of DNA plasmids. These complexes enter NK cells through a unique endocytic pathway, increasing plasmid DNA transfection efficiency from the very low levels typical of conventional liposomes to over 40%, thus overcoming a key bottleneck of lipid-based delivery.

Furthermore, to enhance targeting accuracy, environmentally responsive “smart” carriers have emerged as a research focus. Jain et al. [67] and Wang et al. [68] developed nanosystems capable of responding to local tumor conditions such as low pH or elevated enzyme activity, significantly improving the bioavailability and safety of CAR-NK and CAR-T cells at the target site.

Overall, non-viral vector systems—with their high flexibility, safety, and scalability—are overcoming many of the limitations inherent to viral vectors, establishing themselves as a crucial bridge from basic research to the next generation of clinically viable CAR-cell therapies [36, 38].

### Comparative analysis of delivery platforms

#### Persistence versus controllability: the trade-off of clinical benefits based on disease models

The durability-versus-controllability balance represents a core consideration in vector selection. Bot et al. [47] emphasized that the genomic-integration capability of viral vectors offers irreplaceable value for treating refractory hematological malignancies, where a single administration can establish a long-lived CAR-cell population and provide sustained immune surveillance. However, this persistence also introduces challenges for long-term toxicity management. For instance, targeting B-cell antigens can lead to permanent B-cell aplasia, necessitating lifelong immunoglobulin replacement. Moreover, in solid-tumor settings, permanent CAR expression may result in irreversible “on-target, off-tumor” toxicity if the target is expressed at low levels in healthy tissues [69, 70].

It is within this context that non-viral systems, especially mRNA/LNP platforms, highlight their complementary value as a “safety-switch” strategy. Their transient and reversible expression profile offers a distinct approach to overcoming limitations inherent to viral vectors. First, in solid-tumor therapy, transiently expressed CAR cells naturally decline after eliminating tumor cells, thereby minimizing the risk of long-term off-tumor toxicity [71]. Second, the “hit-and-run” mode is particularly advantageous in non-oncological applications—such as treating myocardial fibrosis or clearing infectious pathogens—where the goal is short-term clearance of pathological targets rather than establishing lifelong immunity. Here, transient CAR-T or CAR-M cells can be rapidly cleared after fulfilling their task, significantly reducing risks of acute toxicities like CRS and avoiding prolonged disruption of immune homeostasis [72, 73].

Furthermore, in anti-infective applications—such as generating CAR-M cells in vivo via peptide-modified LNPs to eliminate *Staphylococcus aureus*—transient expression demonstrates rapid responsiveness, safety, and controllability in acute infection settings. This tunable persistence expands the therapeutic scope of CAR therapy beyond oncology to encompass autoimmune disorders, fibrotic diseases, and infectious diseases [74].

#### Transformation barriers and industrialization prospects: the leap from GMP manufacturing to standardized synthesis

From the perspective of manufacturing and clinical translation, the technical barriers and cost-effectiveness profiles differ significantly across delivery platforms. Viral vectors—particularly clinical-grade lentiviral vectors—involve complex production processes, long lead times, high costs, and challenges in ensuring batch-to-batch consistency, all of which limit their widespread adoption [75]. In contrast, non-viral systems, especially chemically synthesized LNPs, offer advantages in standardized production, easy scalability, and lower relative cost, making them increasingly attractive for clinical translation [67].

Moreover, mRNA-based platforms afford exceptional flexibility. Sequence design and synthesis are rapid and modular, allowing for swift iterative optimization of CAR constructs or target switching. Nevertheless, non-viral and mRNA platforms also face distinct challenges: in vivo delivery efficiency remains suboptimal in certain cell types, and innate immune responses to vector components or exogenous mRNA can still be triggered [76].

In summary, the future advancement of in vivo CAR-cell therapy will rely on a deeper understanding and strategic integration of these diverse delivery platforms (Table 1). By adopting a tailored, context-driven selection strategy, the field can move toward more individualized, precise, and safer therapeutic outcomes.

**Table 1:** In vivo CAR delivery platform summary

Carrier Type	Delivery Method	CAR-Cell Product	Target Antigen	Effect	Refs
Cardiolipin-mimetic LNP (CAMP-LNP)	Intravenous injection	Anti-uPAR CAR-T	uPAR	Liver fibrosis collagen area ↓twofold (30 μg/mouse ×4 doses)	[18]
Retrovirus	Intravenous infusion	Autologous anti-CD19-CAR-T	CD19	64% CAR +; B-cell depletion ≥ 39 weeks	[41]
Retrovirus	Intravenous infusion	Autologous CD19-CAR-T	CD19	CD28ζ group: 1285 copies/μg DNA at 2 weeks vs. 26 (P < 0.0001)	[42]
Lentivirus	Ex vivo transduction + infusion	Human anti-HER2-CAR-M	HER2	90% clearance at 48 h (E:T = 10:1); median survival 88.5 d vs. 63 d	[48]
Lentivirus	Ex vivo transduction + infusion	Rhesus/human anti-CD20-CAR-NK	CD20	Transduction efficiency 60%; tumor burden reduced threefold by day 21	[51]
Lentivirus	Single intraperitoneal injection	Human CD8 + anti-CD19-CAR-T	CD19	Peritoneal CAR +30%–50%; B cells < 0.5%	[52]
Adenovirus	Single intravenous injection	Human anti-CD4-CAR-T	CD4	9% CAR +; CD4 cleared in 2 weeks; tumor reduced 90% by day 25	[58]
Antibody-conjugated LNP (Ab-LNP)	Intravenous injection	Anti-CD19 CAR-T	CD19	90% B-cell depletion (2 mg/kg dose, 36 h)	[62]
PS-targeted LNP (PS-sLNP)	Intratumoral injection	Anti-GP75 CAR-M	GP75	Complete tumor regression in 4/6 mice (10 μg/dose ×3)	[63]
DOPS-LNP	Intravenous injection	Anti-FAP CAR-M	FAP	Ejection fraction ↑18% (2 mg/kg ×6 doses, 2-week delayed treatment)	[64]
CD89-LNP (MT-302)	Intravenous injection	Anti-TROP2 CAR-M	TROP2	Tumor growth inhibition 60% (1 mg/kg ×5 doses, Q4D)	[65]
Polymeric nanoparticle (PBAE)	Intravenous injection	In situ anti-CD19-CAR-T	CD19	Mouse leukemia model: Complete tumor regression ≈100%, comparable to ex vivo virus-transduced CAR-T efficacy	[66]
Ionizable LNP	Intravenous injection	In situ anti-FAP-CAR-T	FAP (cardiac fibrosis)	Mouse heart failure model: Cardiac function recovery > 60%, fibrosis area reduced ≈50%	[67]
Gelatinase-responsive nanoparticle (mPEG-PCL)	Intraperitoneal injection	Switchable anti-HER2-CAR-T	HER2	Mouse gastric cancer model: Tumor inhibition ≈85% with CAR-T/NanoSwitch group; serum IL-6 peak only 1/3 of FreeSwitch group	[68]

## The core challenge of the in vivo treatment bottleneck

CAR cell therapy holds significant promise, yet it faces multiple biological and engineering challenges. Although CAR-T, CAR-NK, and CAR-M therapies share the core logic of target-mediated activation, their distinct cellular biologies endow them with different strengths and limitations when confronting key therapeutic bottlenecks. Focusing on four central challenges—cell persistence and survival, tumor heterogeneity and on-target safety, physical and immune barriers in solid tumors, and the precision of in vivo editing—this section will systematically analyze the response strategies of each cellular platform. By integrating their complementary advantages, we aim to outline a convergent strategy and future pathway toward the next generation of intelligent, in vivo-engineered immunotherapies.

### Expansion, survival, and exhaustion challenges

In vivo CAR-cell therapy is transitioning from the precision manufacturing paradigm of ex vivo preparation toward a new in situ synthesis model, in which immune cells are directly engineered within the patient’s body. While promising, this shift highlights a critical bottleneck: CAR-cells generated via in vivo delivery exhibit markedly poorer survival and persistence compared to their ex vivo-manufactured and infused counterparts [77, 78]. The fundamental challenge lies in replicating high-quality cellular products—achievable only under precisely controlled in vitro conditions—within the complex, dynamic, and often hostile in vivo microenvironment [19, 79]. This constraint directly compromises the long-term antitumor efficacy of the therapy and increases the risk of tumor relapse. The underlying causes are multifaceted and interconnected, which can be primarily attributed to the following three levels.

#### The contradiction between signal strength and persistence

The mRNA-based CAR expression platforms have emerged as a promising strategy for non-viral gene delivery and in vivo CAR-cell therapy, primarily owing to their capacity for transient protein expression and the associated safety advantages [80]. However, the clinical translation of this approach is constrained by a central challenge: the inherent instability of conventional linear mRNA in cells—with a typical degradation rate constant ${k}_{deg\_trans}$estimated at 0.037 h⁻^1^—results in a rapid decline of CAR protein expression, typically weakening substantially within days after a single dose [78]. This transient expression profile struggles to provide the sustained costimulatory and cytokine signaling (CD28, 4-1BB, IL-2) necessary for long-term functional persistence of CAR cells in vivo [81, 82]. Consequently, current linear mRNA platforms generally face an inherent trade-off between signal strength and expression durability, which can lead to under-activated or prematurely exhausted CAR cells and thereby limit their antitumor efficacy in vivo [83].

#### The absence of cytokines and costimulatory signals

In vitro culture systems can simulate an optimal immune-response environment, providing key “nutritional support” for immune-cell expansion, memory phenotype induction, and delayed functional exhaustion through high concentrations of cytokines (IL-2, IL-7, IL-15) and optimized co-stimulatory signals [84, 85]. In contrast, CAR cells generated via in vivo delivery systems often fail to access a similarly supportive microenvironment. Under physiological conditions, the body typically cannot sustain the high levels of cytokines and co-stimulatory signals required for prolonged CAR-cell activation, proliferation, and survival [86].

Furthermore, the local or systemic environment into which CAR cells are introduced is frequently shaped by pathological contexts that impair CAR-cell functionality. In the TME, inhibitory molecules (PD-L1, adenosine) and regulatory immune cells actively suppress CAR-cell activity and accelerate exhaustion [87–89]. In autoimmune settings, although classic tumor-like immunosuppression may be absent, elevated levels of inhibitory cytokines (TGF-β, IL-10), pro-apoptotic factors (e.g., Fas ligand), and dysregulated immune-regulatory networks similarly hinder CAR-cell expansion, promote apoptosis, and hasten functional decline [90–92].

Thus, in vivo-generated CAR cells confront a dual challenge: they lack the exogenous cytokine and co-stimulatory support available in vitro, while simultaneously facing inhibitory pressures from the pathological microenvironment—be it tumor-related or autoimmunity-driven. Together, these factors lead to limited expansion, reduced survival, insufficient memory subset formation, and impaired resistance to exhaustion, ultimately compromising therapeutic durability and efficacy [34, 93].

#### Poor-quality seeds in hostile soil

Immunocytopenia and systemic immunosuppression are common in patients following multiple lines of therapy [94, 95]. T-cell exhaustion is characterized by high expression of inhibitory receptors (PD-1, CTLA-4, TIM-3, LAG-3), which diminishes cytokine secretion and proliferative capacity [96, 97]. Similarly, exhausted NK cells exhibit elevated expression of inhibitory receptors (PD-1, TIGIT, TIM-3, NKG2A) alongside downregulated activating receptors (NKG2D, DNAM-1) [98, 99]. Furthermore, M2-polarized macrophages not only promote tumor growth, angiogenesis, immunosuppression, and tissue repair through secretion of IL-10, TGF-β, and VEGF, but also directly suppress the function of T cells and NK cells [100–102]. These conditions collectively yield a limited pool of CAR-cell “seeds” that are both quantitatively insufficient and qualitatively impaired. The adverse basal host state and the mutually inhibitory microenvironment together form a “wall of survival” that must be surmounted—a major barrier to achieving durable functional persistence of in vivo-generated CAR cells.

### Tumor heterogeneity, off-target and over-activation challenges

While overcoming the limitations of traditional allogeneic infusion, in vivo CAR-cell therapy confronts a more complex targeting paradox. Spatiotemporal tumor heterogeneity, diverse antigen-expression profiles, and the varied composition of immune and stromal cells in the microenvironment collectively create a dynamic and hazardous “minefield.” This challenge directly impacts not only therapeutic efficacy (antigen escape) but also safety (off-target toxicity) and controllability (over-activation). The core dilemma lies in the fact that CAR cells generated in situ must accurately identify and eliminate moving, evolving targets—including those that share antigens with healthy tissues—without the benefit of ex vivo quality checks and dose calibration. When this precision fails, consequences range from therapeutic inefficacy to life-threatening adverse events. This challenge manifests at two interrelated levels.

#### The dual failure risk driven by heterogeneity

Advances in spatial transcriptomics and single-cell sequencing have systematically revealed the highly complex and dynamic spatiotemporal heterogeneity of tumor antigens [103–105]. This heterogeneity poses significant challenges for in vivo CAR-cell therapy. First, antigen expression exhibits pronounced anatomical and functional variation. Antigen profiles differ across tissues and even within the same tumor—between hypoxic/necrotic cores, metabolically active invasive fronts, and immune-cell-rich regions—requiring CAR cells to adapt to local microenvironments and precisely target tumor subpopulations with distinct antigenic characteristics [106–108]. Second, tumor antigens are not static but evolve under therapeutic pressure. Pre-existing subclones with low or absent target antigen expression may expand after CAR-mediated clearance of antigen-positive cells, leading to antigen escape via epigenetic regulation, antigen loss, or modification, and ultimately to disease relapse [109, 110]. Furthermore, spatiotemporal heterogeneity heightens off-target risks. Many tumor-associated antigens (TAAs) are also expressed at low levels or in specific spatiotemporal patterns in normal tissues, which can result in simultaneous tumor attack and healthy-tissue injury [111, 112].

For CAR-T cells, this dynamic can drive an immunoediting process of “antigen-positive cell clearance—selective expansion of antigen-negative clones,” eventually causing relapse. Examples include antigen loss or downregulation in CD19 CAR-T therapy for B-cell acute lymphoblastic leukemia (B-ALL) [113], and lineage switching toward monocyte/macrophage-like phenotypes under CD19 CAR-T pressure [109]. In BCMA CAR-T therapy for multiple myeloma, high levels of soluble BCMA or adjacent membrane proteins (CD55, CD59) can create steric hindrance, leading to epitope masking or modification [114]. Critically, ideal tumor-specific antigens (TSAs) are rare in solid tumors; CARs typically target TAAs that are physiologically expressed at low levels in some normal tissues. Although in vivo-generated CAR-T cells may exhibit weaker expansion and persistence than their ex vivo counterparts, their systemic distribution still poses a risk of severe, even fatal, “on-target, off-tumor” toxicities—such as hepatotoxicity and lung epithelial injury [115, 116].

CAR-M therapy faces distinct challenges. While macrophages naturally infiltrate tumors, the TME contains a spectrum of macrophage phenotypes, from anti-tumor M1 to pro-tumor M2 states [117, 118]. Although CAR-M can enhance phagocytosis and antigen presentation, uneven antigen expression across the TME may limit its ability to reprogram all tumor-associated macrophages (TAMs). Local factors could even cause CAR-M to revert to a pro-tumorigenic phenotype [119]. Off-target risks remain serious: antigens such as HER2, EGFR, and PSMA are also expressed on tissue-resident macrophages (alveolar macrophages, Kupffer cells) that maintain homeostasis. Targeting these antigens may impair critical defense and repair functions—for example, HER2-targeted CAR-M could attack alveolar macrophages and low-HER2-expressing cardiomyocytes [120].

CAR-NK cells possess inherent mechanisms for self-recognition through multiple activating (NKG2D, CD16) and inhibitory receptors (KIRs, NKG2A), theoretically lowering their off-target risk compared to CAR-T cells [121, 122]. However, tumor heterogeneity can impair efficacy via a different mechanism: NK-cell activity is regulated by a balance of signals. If tumor cells downregulate activating ligands (MICA/B) and upregulate inhibitory ligands (HLA-E, PD-L1), even CAR signaling may fail to fully activate CAR-NK cytotoxicity, resulting in incomplete clearance of heterogeneous tumors [123, 124].

####  Systemic risk of “over-activation” in vivo

In ex vivo CAR-cell therapy, the infused cell dose can be partially controlled [113, 125]. In contrast, the active cell dose in vivo CAR therapy depends on in situ generation and expansion, which is influenced by variables such as delivery efficiency, host immune status, and antigen burden, making it difficult to predict or regulate precisely [126, 127].

For CAR-T cells, over-activation primarily manifests as CRS and immune effector cell-associated neurotoxicity syndrome (ICANS) [128, 129]. For example, in CD19-targeted CAR-T therapy for acute lymphoblastic leukemia, excessive tumor burden has triggered explosive CAR-T expansion leading to fatal cerebral edema from severe CRS [130]. Similarly, in BCMA-targeted CAR-T therapy for multiple myeloma, persistent activation within the bone marrow has induced severe ICANS, including seizures and impaired consciousness [131]. Notably, a preclinical study on in vivo CAR-T generation observed that repeated high-dose intravenous administration of targeted nanoparticles (5 × 3 × 10^11^ particles) elevated serum IL-6 levels 2.5-fold—even when delivering a non-specific control CAR gene [132]. This indicates that if the delivery system causes widespread CAR gene modification in immune cells or high target-antigen expression in lymphoid tissues, the intrinsic immunogenicity of the vector may amplify CAR-T over-activation and exacerbate systemic toxicity.

CAR-M over-activation presents a distinct toxicity profile, centered on risks of chronic tissue damage and fibrosis [133, 134]. Preclinical studies show that HER2-targeted CAR-M can induce myocardial inflammation and upregulate pro-fibrotic TGF-β [52]. In pulmonary fibrosis models, adoptive transfer of activated macrophages significantly increases collagen deposition [135]. Moreover, as professional antigen-presenting cells, CAR-M may trigger autoimmunity via epitope spreading after phagocytosing healthy tissues—a risk analogous to anti-MOG antibody-associated demyelination observed in CD19-targeted CAR-T therapy [136]. Thus, CAR-M over-activation warrants particular attention to chronic inflammatory injury, fibrosis, and autoimmune sequelae.

Compared to CAR-T and CAR-M, CAR-NK cells carry a lower risk of severe CRS due to their distinct and more limited cytokine secretion profile [137, 138]. However, over-activated CAR-NK can still cause systemic side effects such as fever and elevated liver enzymes via abundant IFN-γ release [139]. A unique risk lies in their heightened potential to attack healthy cells that express low levels of the target antigen and lack HLA class I molecules—normally a source of NK inhibitory signals [140, 141].

In summary, tumor heterogeneity, off-target toxicity, and over-activation together form a “triple gate” that must be navigated for the safe and effective application of in vivo CAR therapy. Addressing these challenges will require next-generation intelligent programming strategies, including logic-gated CARs (“AND-gate” designs for improved specificity) [142], microenvironment-responsive promoters (hypoxia-inducible factor-1α) [143], and integrated safety modules such as kill switches [144] or molecular brakes (iCAS9) [145]. Concurrently, combining multi-omics-based patient stratification for personalized target selection [146] with local radiotherapy or immunomodulators to remodel the tumor microenvironment and homogenize antigen expression [147, 148] will be key to overcoming these barriers and unlocking the full potential of in vivo CAR-cell therapy.

### Physical barriers and immunosuppressive microenvironment in solid tumors

#### Solid tumor physical barrier

The physical barriers of solid tumors present a primary obstacle to the effective delivery and infiltration of CAR-cell therapies [149]. These barriers are composed of dense extracellular matrix (ECM), abnormal tumor vasculature, and elevated interstitial fluid pressure, which collectively form a restrictive microenvironment [150–152].

For CAR-T cells, their large size and dependence on chemokine-guided migration (CXCL9/10/11–CXCR3 axis) limit their ability to penetrate dense stroma and uniformly infiltrate the tumor core, often restricting therapeutic activity to peripheral regions [153, 154]. CAR-M possess greater inherent tissue-infiltration capacity and can remodel the ECM through secretion of matrix metalloproteinases (MMPs) [155]. However, excessive activation may lead to non-selective matrix degradation (via MMP9) that could inadvertently create channels for tumor dissemination, thereby increasing metastatic risk [156, 157].

CAR-NK cells are relatively small and theoretically more penetrative. However, Wolf et al. observed that tumor-infiltrating NK cells exhibit larger and more irregular morphology compared to circulating NK cells, suggesting physical adaptation—and potential resistance—during tissue entry [158]. Moreover, CAR-NK cells remain constrained by irregular perfusion and poor oxygen/nutrient supply in hypoxic tumor cores [159]. Importantly, physical barriers not only hinder cell entry but also impede the diffusion of critical effector molecules such as cytokines (IL-2, IFN-γ) and chemokines (CXCL12), further dampening antitumor responses [150, 152].

To overcome these fundamental limitations, in vivo CAR-cell therapy offers a promising alternative: the direct, targeted delivery of gene-editing vectors (lentiviral/adeno-associated viral vectors or targeted mRNA-LNPs) to endogenous immune cells—such as tumor-infiltrating lymphocytes, tumor-associated macrophages, or NK cells—that have already been recruited to the tumor site or microenvironment. This approach bypasses the need for ex vivo expansion and systemic infusion, instead reprogramming resident immune cells in situ to mount an immediate, localized attack from within the tumor. Theoretically, this strategy could more effectively overcome physical barriers and enhance therapeutic penetration [69, 127].

#### Immunosuppressive microenvironment

The immunosuppressive TME of solid tumors represents a central obstacle to achieving durable antitumor activity with CAR-cell therapies. This environment is enriched with immunosuppressive cells—such as regulatory T cells (Tregs) and TAMs (predominantly M2-polarized)—and characterized by high levels of inhibitory factors (TGF-β, IL-10) and elevated expression of immune-checkpoint molecules (PD-L1/PD-1, CTLA-4), forming a multilayered inhibitory network [160, 161].

For CAR-T cells, sustained exposure to TCR and PD-1 signaling in the TME readily induces functional exhaustion, accompanied by upregulation of exhaustion-associated transcription factors such as TOX and epigenetic reprogramming. Even when CAR-T cells successfully infiltrate the tumor, their proliferative capacity and effector functions are often rapidly attenuated [140, 162–164]. While CAR-T cells can establish long-lasting memory responses in hematologic malignancies, the inhibitory milieu of solid tumors (TGF-β and IL-10) severely impairs the formation and maintenance of such memory [165, 166].

In contrast to the perforin- and granzyme B-mediated rapid apoptosis induced by CAR-T cells [167], CAR-M exhibit a distinct functional profile in this setting. Their core mechanism involves the phagolysosomal degradation pathway: upon target recognition, activated Syk-kinase signaling drives cytoskeletal rearrangement to form a phagocytic cup, enabling engulfment of entire tumor cells, followed by complete degradation within acidic phagolysosomes [156]. This process not only physically eliminates tumor cells but also generates abundant antigen fragments, positioning CAR-M as efficient antigen-presenting cells that can stimulate polyclonal T-cell responses and create a potent in situ vaccine effect [52].

Simultaneously, the unique value of CAR-NK cells has gained increasing recognition. A key advantage of CAR-NK cells lies in their MHC-independent, multimodal cytotoxicity: beyond inducing apoptosis via perforin/granzyme B and death-receptor ligands (FasL, TRAIL), activated CAR-NK cells secrete cytokines such as IFN-γ and GM-CSF to directly modulate the immune microenvironment [168, 169]. Importantly, this MHC-independent nature allows CAR-NK cells to effectively target tumor cells that evade T-cell recognition through downregulation or loss of MHC class I molecules, directly countering a major immune-escape mechanism [170, 171]. This capacity complements CAR-T activity and provides a critical line of defense against tumor heterogeneity.

**Fig. 2:**
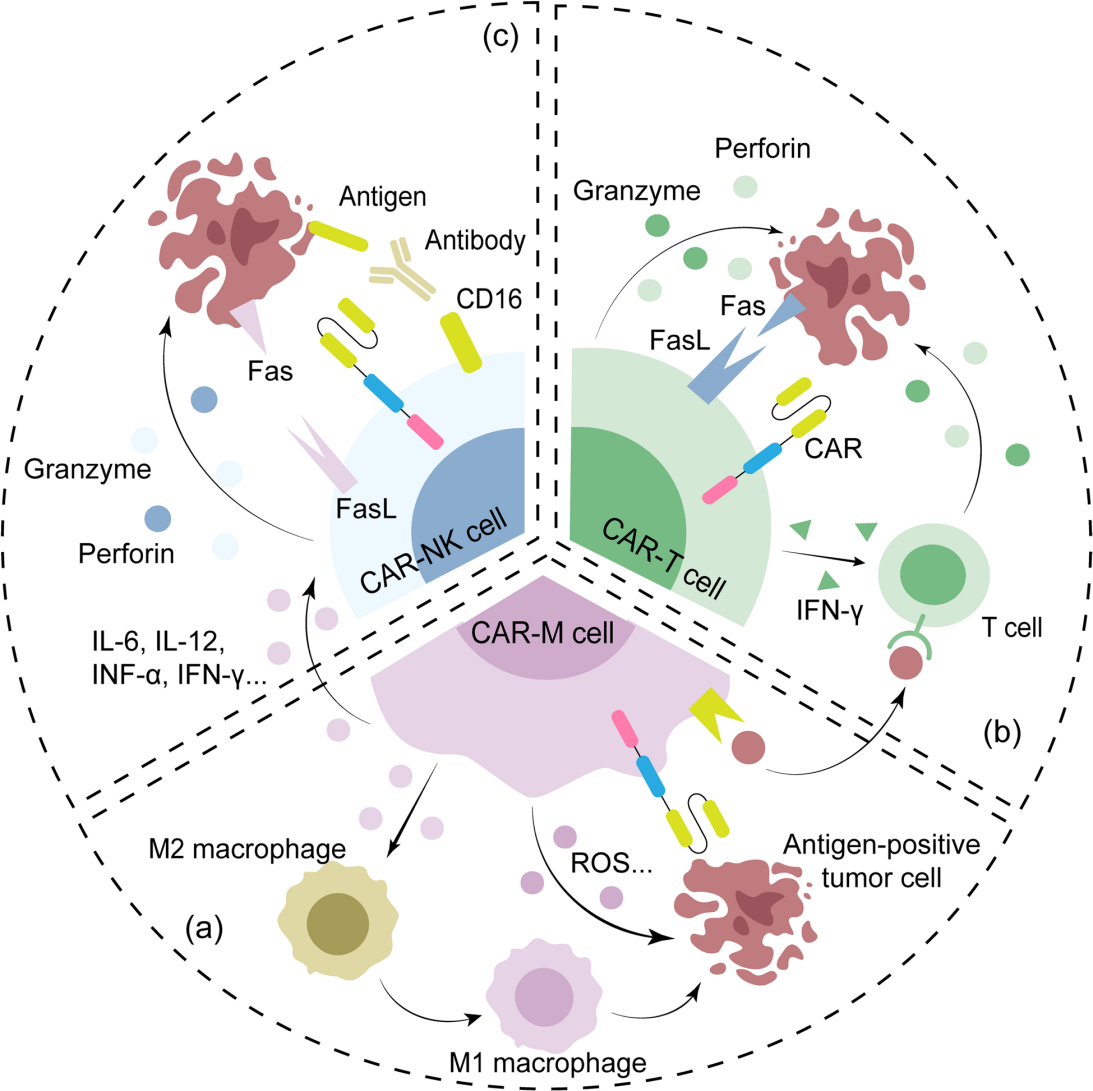
Comparative mechanisms of action of CAR-T, CAR-M, and CAR-NK cells in antitumor immunity. This figure illustrates the complementary mechanisms through which adoptively transferred CAR-T, CAR-M, and CAR-NK cells mediate tumor cell killing within the tumor microenvironment. **a** CAR-M cells exert anti-tumor activity through three key mechanisms: Direct phagocytosis and ROS-mediated cytotoxicity against tumor cells; enhanced antigen presentation that promotes T-cell activation; immune microenvironment remodeling via secretion of cytokines such as IL-6, IL-12, TNF-α, and IFN-γ, which activate T and NK cells and polarize macrophages toward the pro-inflammatory M1 phenotype. **b** CAR-T cells exert anti-tumor effects through multiple synergistic mechanisms. They directly induce apoptosis in antigen-expressing tumor cells via perforin and granzyme B release. Additionally, CAR-T cells secrete cytokines such as IFN-γ, activating both themselves and bystander T lymphocytes in the tumor microenvironment, thereby amplifying the local immune response. Furthermore, through the Fas/FasL signaling pathway, CAR-T cells can also eliminate tumor cells that lack the target antigen, helping to overcome challenges posed by tumor heterogeneity. **c** CAR-NK cells engage tumor cells through antigen-specific recognition via their chimeric antigen receptor (CAR). Simultaneously, the native activating receptor CD16 (FcγRIII) binds to the Fc portion of tumor-bound IgG antibodies, initiating antibody-dependent cellular cytotoxicity (ADCC). Upon activation, CAR-NK cells exert direct cytotoxic effects through the release of perforin and granzyme B, which induce apoptosis in the target tumor cell

Within this framework, different CAR-cell platforms collectively form a functionally synergistic “synthetic immune system” (Fig. 2): CAR-M acts as a TME remodeler and immune-response initiator, breaking immunosuppression and triggering broad immune activation. CAR-T serves as a precise cytotoxic effector and memory-forming population, enabling targeted clearance and sustained immune surveillance within a remodeled microenvironment. CAR-NK operates as an MHC-independent, rapid-response unit, delivering immediate cytotoxicity and filling gaps in immune recognition. Together, they offer a layered and adaptable strategy to overcome the multifaceted barriers of solid tumors.

###  Precise targeting and safety of in vivo editing

The emergence of in vivo editing technologies signals a paradigm shift in CAR-cell therapy, moving from a “cell-as-a-drug” model toward a “patient-as-a-bioreactor” framework. The central challenge of this revolutionary approach lies in ensuring that gene-editing instructions are delivered with precision, efficiency, and safety exclusively to the intended immune-cell populations, while mitigating risks such as off-target editing, vector-associated toxicity, and uncontrolled immune activation. Addressing this challenge extends beyond technical feasibility; it will fundamentally shape the evaluation of different immune cell types as “ideal editing substrates” for future therapeutic development.

#### Targeted delivery, editing efficiency, and specificity

Targeted delivery and cell-type-specific editing efficiency remain the primary obstacles to achieving precise in vivo reprogramming. Success largely depends on the tropism of the delivery vector for particular immune cells and its efficacy in intracellular delivery [172–174].

Currently, myeloid cells such as macrophages demonstrate clear advantages. Monocytes and macrophages are professional phagocytes with a strong intrinsic capacity to internalize nanoparticles and retain them for extended periods. For instance, studies show that model macrophages can uptake silica nanoparticles at rates up to 7.9 × 10^4^ particles/hour/cell, with approximately 80% of particles retained intracellularly 48 h after internalization [175]. Formulation engineering of LNPs—by tuning lipid composition, surface charge, and ligand display—enables efficient targeting of myeloid cells in vivo, as demonstrated by the preferential accumulation of intravenously administered LNPs in macrophages within the liver, spleen, lungs, and tumors [176, 177]. This inherent affinity makes in vivo generation of CAR-M technologically more straightforward.

In contrast, editing circulating T cells and NK cells presents greater challenges. As non-phagocytic lymphocytes, T cells are notoriously difficult to transfect with non-viral vectors [178, 179]. Current strategies rely on conjugating antibodies or ligands against T-cell surface markers (CD3, CD4, CD5, CD8) onto LNPs [73, 180, 181]. However, the functional and phenotypic heterogeneity among T-cell subsets (naïve vs. effector T cells) and across differentiation stages complicates uniform editing and raises concerns about disrupting normal T-cell homeostasis [40, 146, 182, 183].

NK cells, on the other hand, offer notable potential as editing substrates. While also circulating in blood, they express unique surface markers (NKG2D, CD56) that can be exploited for specific targeting [184, 185]. Moreover, certain NK-cell subsets (CD56bright) exhibit modest endocytic activity, which may facilitate LNP uptake [186]. Their inherent safety profile—including a lower risk of cytokine release syndrome—means that variability in editing efficiency or minor off-target effects may be clinically more manageable compared to T-cell-based approaches [138].

#### Differential tolerance of cell substrates

The safety risks associated with in vivo editing vary substantially across different immune cell types, directly influencing their suitability as editing substrates and defining the upper limit of acceptable clinical risk [187].

Among these, targeting macrophages entails a prominent risk of vector-induced immunogenicity. As central mediators of inflammatory responses, macrophages can amplify the innate immune reaction triggered by LNP-mRNA delivery, potentially leading to unintended local or systemic inflammation [188, 189]. In contrast, T-cell and NK-cell responses to similar stimuli remain less characterized but are anticipated to differ in their effect profiles.

At a more fundamental level, the risk of off-target genome editing is most severe in T cells, given their potential for malignant transformation and the consequent risk of secondary malignancies [182, 190, 191]. For NK cells, the limited lifespan and proliferative capacity substantially reduce the likelihood of long-term clonal expansion resulting from editing errors [192]. Although terminally differentiated macrophages carry minimal oncogenic risk, off-target editing in these cells may disrupt phagocytic function, polarization, or secretory activity, thereby impairing tissue homeostasis and potentially promoting fibrotic pathways [52].

These cell-type-specific behavioral risks form a critical framework for evaluating an “ideal editing substrate.” For in vivo-generated CAR-T cells, stringent control is required to prevent CRS and immune effector cell-associated neurotoxicity syndrome (ICANS) driven by persistent activation. CAR-NK cells offer a wider therapeutic margin owing to their intrinsically favorable safety profile, while CAR-M demand precise calibration of activation thresholds to avoid excessive inflammation and tissue damage.

In summary, from a safety-tolerance perspective, NK cells exhibit distinct advantages. Their higher safety threshold provides a more robust risk buffer, positioning them as a particularly promising substrate for in vivo editing platforms.

####  Who is the ideal “in vivo editing substrate”?

A comprehensive evaluation of targeting feasibility, safety profile, and therapeutic potential reveals distinct trajectories for the three major immune-cell types as future substrates for in vivo editing. Macrophages—with their inherent phagocytic activity, efficient nanoparticle uptake, and low oncogenic risk—are most compatible with existing LNP delivery platforms, representing the most feasible near-term translation pathway. The central challenge lies in precisely regulating their potent effector functions to avoid excessive inflammation and tissue damage. NK cells strike a favorable balance between targetability, editing safety, and therapeutic function. Their “plug-and-play” characteristics—including independence from complex co-stimulation, multimodal cytotoxicity, and an intrinsically favorable safety profile—position them as an ideal platform for mid-term, broadly applicable in vivo therapies, capable of generating safe and effective fighters with relatively modest engineering. T cells, despite facing formidable targeting hurdles and higher oncogenic risk, remain the ultimate therapeutic ambition due to their unparalleled capacity for clonal expansion and long-term immunological memory. Overcoming their editing bottlenecks will require disruptive technological advances; success in this endeavor would mark a pinnacle achievement in the field of in vivo cell engineering.

## The roadmap of in vivo editing: Strategies for synergistically enhancing the efficacy of CAR cells and the clinical challenges involved

### Multi-dimensional collaboration to break through the bottlenecks of cell survival and persistence

Faced with the multiple challenges of expansion, survival, and exhaustion resistance in in vivo CAR-cell therapy, current research and development efforts are integrating precision genetic engineering and advanced delivery technologies to equip effector cells with a competitive survival advantage within complex pathological environments (Table 2). The core of this technological evolution lies in establishing a “three-in-one” multidimensional collaborative framework. First, ligand-guided capture and spatiotemporal targeting enable the pre-enrichment of editing tools in specific immune subsets. Second, innovations in nucleic-acid vector design and expression architecture support efficient and sustained transgene production, providing the molecular foundation for durable activity. Finally, remodeling key signaling pathways enhances effector-cell function and phenotypic fitness, endowing cells with endogenous resilience against immunosuppressive microenvironments.

**Table 2:** An overview of collaborative delivery strategies to break the bottleneck of cell survival and persistence

Carrier Type	Delivery Method	CAR-Cell Product	Target Antigen	Effect	Refs
Cardiolipin-mimic LNP	Intravenous	In vivo generated CAR-T cells (senolytic)	uPAR	Alleviated liver fibrosis and rheumatoid arthritis in mice	[18]
Lentivirus (targeted)	In vivo infusion	BCMA-CAR-T (ESO-T01)	BCMA	CAR-T detected in blood and tumor tissues for up to 2 months; partial remission in myeloma	[36]
CD3-targeted LV	Intravenous injection	Anti-CD19 CAR-T	CD19	Efficient and selective transduction of T cells in whole blood; CAR + T cells generated in vivo; CD19 + B cells depleted in humanized mice	[49]
CRV-modified LNP	Intravenous injection	Anti-SasA CAR-M	SasA (S. aureus surface protein A)	Enhanced MRSA clearance, improved survival in sepsis mice	[72]
CD4-targeted LV	Intravenous injection	Anti-CD19 CAR-T	CD19	Selective CAR delivery to CD4 + T cells in vivo; enhanced tumor clearance compared to CD8 + CAR-T; durable B cell depletion in huNSG mice	[193]
CDV Adapter-LV	Intravenous injection	Anti-CD20 CAR-T	CD20	Adapter-mediated selective transduction of CD4 + and CD8 + T cells in vivo; functional CAR-T generation without prior activation; B cell depletion in PBMC-NSG model	[194]
sEV (anti-CD206 scFv modified)	Inhalation	Anti-MSLN CAR-M	MSLN (mesothelin)	Inhibited lung metastasis & recurrence, prolonged survival in mice	[195]
LNP (mRNA)	Intravenous injection	Anti-HER2 CAR-NK	HER2	Potent tumor killing and cytokine production in NK cells; NKp44-CAR dependent on DAP12 for expression	[196]
NK cell line (NK-92MI)	Ex vivo engineering + infusion	NK-cell Biofactory (NK-92MI)	FRα/MSLN	Antigen-specific cytolysis and calibrated protein secretion in situ; preserves function post-irradiation	[197]
Ionizable LNP (Mannose-coated)	Intracapsular injection	Anti-CA9 CAR-M + CSR	CA9	Tumor growth inhibition > 80%, survival extension to 60 days in renal cancer model	[198]
Retrovirus	Intravenous infusion	Autologous 7 × 21 CAR-T cells (IL7/CCL21 armored)	CLDN18.2	Without lymphodepletion, showed superior tumor growth inhibition (up to 98.8%) in solid tumor models	[199]
Retrovirus (Cord blood derived)	Intravenous injection	CAR-NK cells secreting IL-15	CD19	~ 40% cure rate in lymphoma xenografts; early toxicity observed in immunodeficient mice	[200]
Retrovirus	Ex vivo transduction + infusion	BCMA CAR-NK-92 cells co-expressing CXCR4	BCMA	Enhanced migration, cytotoxicity, and synapse signaling against multiple myeloma in vitro	[201]

#### Targeted delivery strategies

The core challenge of in vivo cell-specific editing lies in accurately identifying target immune subsets within the complex physiological milieu while avoiding nonspecific clearance by the reticuloendothelial system (RES) and off-target uptake in healthy tissues [202]. Shi et al. [76] emphasize that advancing from a “wide-net” to a “precision-guided” delivery paradigm—through optimization of ligand architecture and conjugation chemistry—is essential to achieve specific receptor engagement and improve the therapeutic index [67].

In the field of T-cell reprogramming, vector surface engineering has reached a mature stage. Frank et al. [49] developed an anti-CD3 scFv-displaying lentiviral vector (CD3-LV) that achieved up to 40% transduction of resting T cells in vivo. Subsequent work established protocols for generating CD4^+^- or CD8^+^-specific CAR-T subsets [203]. For instance, Agarwal et al. [193] used a CD4-targeted LV to produce Th1/Th2-polarized CAR-T cells with sustained persistence in vivo, while Winter et al. [194] designed a modular adapter-LV system for flexible targeting. On the non-viral front, Liu et al. [204] employed CD3-directed LNPs to deliver CAR mRNA and successfully restored function in damaged myocardial tissue.

For innate immune cells, Yang et al. [205] engineered LNPs that adsorb plasma proteins to selectively target liver-resident macrophages. Xiao et al. [195] developed inhalable exosomes (sEVs) displaying anti-CD206 scFv to reprogram pulmonary M2 macrophages and suppress lung metastasis. Similarly, Tang et al. [72] used peptide-modified LNPs to generate *Staphylococcus aureus*-targeted CAR-M cells in situ. In the NK-cell domain, Diwanji et al. [196] leveraged natural receptor pathways to achieve specific in vivo activation, and Repellin et al. [197] proposed an “NK-cell bio-factory” concept wherein locally engineered NK cells continuously secrete therapeutic proteins to overcome their limited persistence. Looking forward, Li et al. [36] highlight that integrating environmentally responsive linkers with targeting ligands will be key to enhancing cell-type specificity.

#### Innovative delivery vectors and expression elements

To address the bottleneck of transient transgene expression, the current research and development focus is shifting from short-lived expression toward sustained delivery. A key breakthrough in this transition is the use of circular RNA (circRNA) as an alternative to conventional linear mRNA. The covalently closed, exonuclease-resistant structure of circRNA enables significantly prolonged protein expression in immune cells—often severalfold longer than linear mRNA—providing CAR cells with a more stable and durable molecular “arsenal” [206, 207].

This strategy has already demonstrated promising results. Jing et al. employed circRNA to encode an IL-2R–TLR4 chimeric signaling receptor (CSR) together with a CA9-targeting CAR, effectively activating T-cell immune responses [198]. In another study, Zhang et al. used PL40-formulated lipid nanoparticles to deliver a circRNA-encoded CAR, achieving durable T-cell reprogramming with transfection efficiencies up to 99% and superior clearance of senescent cells compared to linear RNA counterparts [18]. The enhanced stability of circRNA successfully overcomes the short-lived expression limitation inherent to linear mRNA, establishing a practical foundation for developing durable in vivo CAR therapies based on transient genetic delivery.

#### Engineering intrinsic properties of cells

Beyond advances in delivery systems, a third dimension for improving in vivo cell engineering involves modifying the intrinsic properties of immune cells to enhance their survival and function within complex microenvironments. One approach is to directly supplement key survival and proliferative signals through co-expression of critical molecules. For example, engineering CAR cells to co-express cytokines such as IL-7 and IL-15—or their cognate receptors—enables autocrine signaling that sustains expansion and persistence [199, 200]. Similarly, overexpressing the chemokine receptor CXCR4 can promote homing of CAR-NK cells to protective niches like the bone marrow, acting as a “survival sanctuary” [201].

Conversely, actively disrupting inhibitory pathways can equip cells with “exhaustion-resistant armor.” Strategies include expressing a dominant-negative FAS receptor (ΔFAS) to block apoptosis [208] or using CRISPR/Cas9 to knock out PD-1, thereby desensitizing CAR-T cells to suppressive signals in the tumor microenvironment [209]. For CAR-NK cells, eliminating the self-antigen CD7 during the stem-cell stage prevents fratricide and preserves a functional cell pool [210, 211].

In summary, current proactive strategies form a multi-level, synergistic framework: delivery-technology innovations address target recognition, vector-design breakthroughs improve transduction efficiency, and intrinsic cell engineering enhances cellular fitness. Together, these complementary approaches are accelerating the development of in vivo CAR-cell therapies toward a next-generation clinical reality characterized by robust and durable efficacy.

### Early clinical trial data and challenges

With the accumulation of preclinical evidence, various in vivo CAR therapies have entered the stage of early clinical validation, revealing their safety, feasibility, and unique challenges in humans [212–217]. Currently, several CAR-T therapy products have been approved for hematological malignancies, but their in vivo application in solid tumors remains in early-stage exploration. Clinical trials for CAR-NK and CAR-M therapies have just begun, making the relevant data particularly valuable. Despite differences in the targeted cell types, a core translational challenge to in vivo CAR therapies is the effective and safe delivery of these engineered immune cells to patients, enabling durable antitumor activity within the complex physiological environment [127, 218].

A new generation of CAR-T therapy is advancing from ex vivo preparation to in vivo generation. Early clinical trials have preliminarily verified its feasibility [219] while also exposing core bottlenecks (Table 3). Operationally, several studies have achieved rapid effects by simplifying processes: NCT07075185 and NCT06791681 have simplified lentiviral transduction into a “single-infusion” procedure, with peak CAR-T cell levels detectable in the periphery within 6 days—at least 7 days shorter than traditional cycles. NCT06917742 used mRNA-LNP for in vivo translation, further shortening the time to peak and eliminating the risk of viral insertional mutagenesis. Some regimens also incorporate drug-responsive elements (UB-VV400, UB-VV111) to enable regulated in vivo expansion, reducing the incidence of ≥ grade 3 CRS/ICANS while maintaining efficacy. However, challenges are simultaneously highlighted: the first-pass effect of mRNA-LNP in the liver and spleen leads to a tenfold individual variation in CAR-T peak levels [62]; combining multiple targets (CD22-CD19 and BCMA-CD19) may broaden antigen coverage but could accelerate T-cell exhaustion; in NCT06743503 and NCT06528301, peripheral CAR-T retention at 90 days was less than 1% [220]. The termination of early projects MY-M11 and TAK-103 suggests that single-cell or single-target approaches are prone to failure due to insufficient persistence or microenvironmental inhibition, indicating a future need to integrate synergistic modalities such as CAR-M and oncolytic viruses [221]. In summary, while the new generation of CAR-T has established a preliminary paradigm in dose exploration and safety monitoring [222], cell durability, precise delivery, and combination strategy optimization remain the three major challenges that must be overcome for its widespread clinical adoption.

**Table 3:** Progress in CAR-T related clinical trials

Clinical trial ID	Phase	CAR-T product	Study status	Target antigen	Conditions	Refs
NCT06528301	I	UB-VV111 ± Rapamycin	RECRUITING	CD19 (LBCL/CLL)	LBCL/CLL	[220]
NCT07109986	I	UB-VV410	NOT YET RECRUITING	SLE/LN	Systemic Lupus Erythematosus/Lupus Nephritis	NA
NCT07075185	I	KLN-1010	RECRUITING	BCMA (MM)	Multiple Myeloma	NA
NCT07002112	I	LVIVO-TaVec100	RECRUITING	CD19(B-cell)	Relapsed/Refractory B-cell Malignancies	NA
NCT06917742	I	CPTX2309	RECRUITING	CD19 (Healthy)	Healthy Volunteers	NA
NCT06791681	I	ESO-T01	RECRUITING	BCMA (MM)	Relapsed/Refractory Multiple Myeloma	NA
NCT06743503	I/II	UB-VV400 ± Rapamycin	RECRUITING	CD22 (LBCL)	Large B-cell Lymphoma	NA
NCT06691685	I	ESO-T01 Injection	RECRUITING	BCMA (MM)	Multiple Myeloma	NA
NCT06678282	I	JY231 Injection	RECRUITING	CD19 (B-NHL/B-ALL)	B-NHL/B-ALL	NA
NCT06618313	I	JCXH-213	RECRUITING	CD19 (B-NHL)	B-Cell Non-Hodgkin Lymphoma	NA

As CAR-NK therapy transitions from “ex vivo customization” to “in vivo manufacturing”, a key challenge is how to transiently construct a precise, safe, and reproducibly accessible natural killer cell army in the human body via in vivo delivery. Several recent ex vivo clinical trials have provided important insights (Table 4). NCT06539338 first introduced the concept of an “in vivo factory” into the CAR-NK field: a single infusion of an integration-deficient lentivirus carrying a CD20-CAR simultaneously generated both CAR-T and CAR-NK cells in the periphery [223]. The peak level of CAR20^+^ NK cells within 28 days showed a linear correlation with the B-cell clearance rate, confirming the feasibility of the “single-drug, dual-cell” strategy. NCT06010472 [224] used umbilical cord blood-derived CD19-CAR-NK to treat refractory systemic lupus erythematosus (SLE). None of the 36 patients experienced ≥ grade 3 CRS/ICANS [225], demonstrating that allogeneic NK cells can exert rapid effects in autoimmune diseases without preconditioning lymphodepletion. The solid tumor trial NCT05410717 employs a four-target CAR-NK for “target-matched, ready-to-infuse” treatment. The peripheral half-life of CAR-NK cells is approximately 10–14 days, while intratumoral CAR signals persist for up to 4 weeks, suggesting that repeated administration can sustain local effects. NCT04623944 further revealed a “cell kinetics–efficacy” relationship: NKX101 achieved an objective response rate of 42% within 28 days in AML/MDS patients, and its peripheral decay half-life (7 days) correlated with the time to first relapse, indicating that the “expansion window” may be a key rate-limiting factor for efficacy. Future research on in vivo CAR-NK therapy [226] must overcome three key challenges: first, the issue of vector tropism, requiring logic-gating or local delivery technologies to avoid the first-pass effect in the liver and spleen, thereby reducing individual differences in transduction efficiency; second, the contradiction between transient expression and repeated administration, necessitating the design of repeatable dosing regimens while avoiding anti-vector immune responses; third, the problem of functional adaptation—how to achieve cytokine signaling that effectively prolongs NK cell survival without triggering systemic inflammation, through autocrine switches or combined small-molecule delivery in vivo, remains a core scientific question in the dose-optimization stage [227].

**Table 4:** Progress in CAR-NK related clinical trials

Clinical trial ID	Phase	CAR-NK product	Study status	Target antigen	Conditions	Refs
NCT06539338	I	INT2104 (CAR20)	RECRUITING	CD20	B-cell Cancers (NHL, B-ALL)	[223]
NCT06010472	I/II	KN5501 (anti-CD19 CAR-NK)	RECRUITING	CD19	Systemic Lupus Erythematosus	[224]
NCT05092451	I/II	CAR.70/IL15-transduced CB-NK cells	RECRUITING	CD7	Hematological Malignancies	[228]
NCT04050709	I	PD-L1 t-haNK	ACTIVE	PD-L1	Solid Tumors	[229]
NCT05410717	I/II	Multi-target CAR-NK (CLDN6/GPC3/MSLN/AXL)	RECRUITING	CLDN6, GPC3, MSLN, AXL	Advanced Solid Tumors	NA
NCT04623944	I	NKX101 (anti-NKG2DL CAR-NK)	ACTIVE	NKG2D Ligands	AML, MDS	NA

As CAR-M therapy shifts from ex vivo preparation to in vivo generation, a key bottleneck in treating solid tumors is how to efficiently transduce monocyte-macrophages and maintain their anti-tumor function. Recent ex vivo clinical trials offer important references (Table 5). NCT04660929 first advanced in vitro-constructed HER2-CAR-M into clinical practice, verifying the safety and feasibility of its dual “phagocytosis–immunity” mechanism [230], with an ORR of 24% and no ≥ grade 3 CRS. NCT06224738 further explored local intraperitoneal perfusion for treating peritoneal metastasis, aiming to establish an M1-dominated microenvironment within tumors. However, ex vivo preparation faces limitations such as long cycles, freezing sensitivity, and cold-chain dependence. More critically, early project data show that CAR-M function declines rapidly after infusion, with a peripheral survival time of less than 7 days, making it difficult to establish continuous immune surveillance. This ex vivo experience has defined clear engineering boundaries for in vivo development: the effective dose of CAR-M can be compressed to 10^4^ copies/μg DNA, and local administration can amplify efficacy more effectively than systemic administration [231]; meanwhile, co-delivery of cytokine signals can significantly prolong functional persistence. This indicates that transitioning from an “ex vivo factory” to an “in vivo snapshot” is not a simple substitution but requires transforming manufacturing challenges into engineering problems at the levels of targeting, expression, and controllability within delivery systems [232]. Only by achieving precise, coordinated delivery of CAR constructs, polarization signals, and safety switches can in vivo CAR-M truly realize its potential against solid tumors.

**Table 5:** Progress in CAR-M related clinical trials

Clinical trial ID	Phase	CAR-M product	Study status	Target antigen	Conditions	Refs
NCT04660929	I	CT-0508	Active not recruiting	HER2	HER2-positive solid tumors	[217]
NCT03608618	I	MCY-M11	Terminated	MSLN	Ovarian cancer and peritoneal mesothelioma	[233]
NCT05969041	I	MT-302 (MYE Symphony)	RECRUITING	Unknown (Epithelial Tumors)	Epithelial Tumors	[234]
NCT06478693	I	MT-303 ± Atezo/Bev	RECRUITING	GPC3	Hepatocellular Carcinoma	[235]
NCT06562647	I	SY001	RECRUITING	Mesothelin(MSLN)	Ovarian Cancer/Solid Tumors	NA
NCT05007379	I	NA	Unknown	HER2	Breast cancer	NA

Early clinical trial data are gradually validating the feasibility and unique value of different in vivo CAR strategies. CAR-T has the most extensive clinical experience, but its success in solid tumors still requires overcoming delivery and microenvironmental barriers. The first clinical data for CAR-M have verified its safety and immune-remodeling mechanism. CAR-NK has demonstrated excellent short-term safety. Future translational success will depend on the continuous iteration and optimization of delivery platforms, cell-engineering strategies, and combination regimens based on these early insights.

## Conclusion and perspectives

As in vivo CAR therapy advances from proof-of-concept toward clinical translation, the spatiotemporal heterogeneity of tumor-host interactions and variations in host immune baselines have emerged as central constraints on both efficacy and safety. To overcome these barriers, therapeutic strategies are evolving toward a model of precision stratification and intelligent design. This section will systematically outline how biomarker-guided patient stratification [219, 236] and AI-enabled therapeutic design [237, 238] can propel in vivo CAR therapy into a new era of truly individualized and predictable treatment.

### Biomarker-driven patient stratification

The spatiotemporal heterogeneity of the tumor-host interaction stands as a central bottleneck limiting the efficacy of in vivo CAR therapy [239]. Building a prospectively validated, biomarker-driven stratification system is therefore crucial to improving response rates and avoiding ineffective toxicity [240–242]. Accurate mapping of the tumor-antigen landscape constitutes the first layer of patient stratification [243]. While conventional immunohistochemistry or flow cytometry only provide average expression levels, advanced spatial profiling techniques—such as multiplex immunofluorescence (mxIF), imaging mass cytometry (IMC), and spatial transcriptomics—enable single-cell-resolution quantification of antigen density, clonal heterogeneity, and spatial relationships with immunosuppressive cells or cancer-associated fibroblasts [244–246]. The application of spatial proteomics (IMC, CODEX) provides an extensible framework for immunotherapy patient stratification [240, 247] For instance, Qiu et al. [244] employed high-resolution spatial protein mapping in 401 hepatocellular carcinoma samples to define distinct cellular neighborhoods, successfully stratifying patients into microenvironmental patterns with significant prognostic differences. This approach aids in identifying patient subtypes most likely to benefit from specific CAR therapies. A recent peri-tumoral microRNA-CAR study further demonstrated that the ratio of antigen-enriched to immune-cell-deficient areas (A/I ratio) is a superior predictor of objective response compared to the traditional H-score [248]. Additionally, deep sequencing of circulating tumor DNA (ctDNA) can dynamically track antigen-loss mutations or splice variants, offering a 6- to 8-week early warning of immune escape and informing the rational design of sequential strategies such as dual-target CARs or epitope-drifting vaccines [249, 250].

After defining the spatiotemporal distribution of target antigens, the functional heterogeneity of the tumor immune microenvironment (TME) emerges as the next critical determinant of therapeutic response. The classical classification by immune-inflamed/excluded/desert is insufficient for predicting efficacy [251]. The functional mapping at single-cell resolution is required [252–254]. Integrated imaging mass cytometry and spatial transcriptomic analyses reveal that CD8^+^ T-cell density should be further subdivided into stem-like TCF1^+^ precursor-exhausted (Tpex) and terminally exhausted (Tex) subsets [255]. The abundance of Tpex cells shows a strong positive correlation with the peak expansion of CAR-T cells in vivo (ρ = 0.82, *P* < 0.001). For CAR-M therapies, the CD163^+^/iNOS^−^ ratio in M2-polarized TAMs and the activity of the CD47-SIRPα axis are key determinants of phagocytic efficiency [256, 257]. Spatial analyses indicate that when CD47 mean fluorescence intensity exceeds 150 AU and the collagen-barrier thickness is greater than 20 μm, the objective response rate of single-agent CAR-M drops below 10%, highlighting the need to combine CD47 blockade or dominant-negative TGF-βR2 receptors to remodel the TME [258, 259]. For CAR-NK cells, the expression profile of inhibitory molecules—including HLA-E, PD-L1, and TIGIT ligands—should guide therapeutic decisions. Their expression levels can inform whether to knockout the NKG2A receptor (KLRC1) via gene editing [260, 261] or to co-express immunomodulatory molecules such as PD-L1 in universal CAR-NK designs to enhance persistence [262]. Blockade strategies targeting inhibitory axes like TIGIT remain an area for further exploration.

Beyond tumor-intrinsic and microenvironmental features, the host systemic immune baseline is a key variable constraining CAR transduction efficiency, expansion kinetics, and persistence in vivo [263]. A comprehensive assessment incorporating peripheral immune profiles, metabolic-inflammatory states, the commensal microbiome, and genetic background can refine individualized stratification [264, 265]. A recent study in large B-cell lymphoma identified pretreatment C-reactive protein (CRP) ≥ 4 mg/dL and serum ferritin ≥ 400 ng/mL as independent risk factors for predicting severe toxicity and poor progression-free survival following CAR-T therapy [266–268], thereby integrating readily accessible inflammatory markers into risk stratification. Preexisting neutralizing antibodies (NAbs) against viral vectors also present a delivery barrier. For patients with high anti-AAV2 NAbs titers, the use of engineered capsids (escape mutants) or non-viral mRNA-LNP platforms should be considered to maintain adequate transduction efficiency [269–273]. Importantly, host germline genetic variation profoundly influences TME cellular composition and modulates treatment response. Cai et al. [258] systematically identified nearly 3,500 immunomodulatory quantitative trait loci (immunQTLs) regulating the abundance of 54 immune-cell types within the TME through an analysis of over 7,700 samples across 23 cancer types, providing a genetic basis for clinical observations such as the association between the *HLA-DRB1*04:01 allele and the generation of anti-murine scFv antibodies.

Looking forward, the integration of multidimensional data—spanning tumor multi-omics and host immune parameters—will enable the construction of machine-learning-based models to predict treatment response. Such models would provide clinicians with a quantitative decision-support tool for determining whether, when, and how to deploy specific in vivo CAR vectors [274]. Ultimately, a robust predictive framework must simultaneously encapsulate tumor-intrinsic characteristics, local TME states, and the systemic immune baseline [275–278] to achieve a precise, multi-scale portrait of each patient [244, 279, 280].

### Role of artificial intelligence in therapy design and optimization

The intelligent application of artificial intelligence in in vivo CAR therapy is currently centered on the initial construction of a closed-loop system for “target discovery to prognosis monitoring.” Its potential value is beginning to emerge across three key areas.

At the target discovery stage, integrating multi-omics data with graph neural networks has provided early indications that TIM-3/GAL-9 signaling and cDC1 deficiency may be associated with CAR-T exhaustion. NOT-gated CAR designs informed by these insights have shown increased killing activity in vitro [281]. Similarly, candidate neoantigens such as CDH17 and FAP have been identified through large-scale plasma proteome models involving over 50,000 samples, offering validated leads for expanding the target library of solid-tumor CARs [282–284].

In prognostic monitoring, early studies demonstrate that multimodal deep learning models (C-index ≈ 0.70) combining histopathological images, ctDNA dynamics, and cytokine profiles can predict the risk of grade ≥ 3 CRS several weeks in advance. This predictive capacity may support dose adjustment of rapalog-regulated ON-switch CAR therapies [285].

Furthermore, automated pipelines such as “nextNEOpi” use machine learning to continuously rank classical and unconventional neoantigens. When linked with the prognostic scoring systems described above, such tools could enable an iterative framework of “target updating, early risk warning, and dose correction” [286]. Although these approaches still require validation in prospective studies, they outline a testable technological pathway toward scalable, intelligence-driven in vivo CAR therapy.

### Future directions and interdisciplinary integration

In the field of cellular immunotherapy, in vivo CAR therapy is undergoing a profound paradigm shift—from “ex vivo manufacturing and reinfusion” toward “in situ in vivo programming” [73]. The goal is to simplify complex production processes into precise in vivo delivery, thereby significantly improving treatment accessibility [287, 288]. Advances have been made in delivery platforms, effector-cell lineage expansion, and early clinical validation, laying a preliminary foundation for broader application [59]. However, the path to translation still faces several core bottlenecks: insufficient delivery precision leads to hepatic/splenic sequestration and off-target risks [289]; limited persistence, a narrow expansion window, and susceptibility to exhaustion constrain the functional lifespan of engineered cells [290, 291]; and the dense physical structure and immunosuppressive microenvironment of solid tumors remain pervasive barriers [292].

Looking forward, overcoming these challenges will depend on the co-evolution of two parallel directions: integration and intelligence. First, next-generation intelligent delivery systems must integrate logic-gated targeting, microenvironment-responsive activation, long-lived expression elements (circular RNAs), and reversible safety switches to enable more precise, durable, and controllable in vivo programming. Second, therapeutic regimens will increasingly adopt multicellular synergy and combination-based immune remodeling—constructing a “synthetic immune corps” in which CAR-T cells enable precise clearance, CAR-NK cells provide rapid response, and CAR-M remodel the microenvironment, complemented by oncolytic viruses, immune checkpoint inhibitors, and other modalities to systematically overcome solid tumors.

Furthermore, fully individualized treatment will become central to the paradigm. By integrating multidimensional biomarkers for patient stratification and deploying artificial intelligence to assist in target discovery, CAR design optimization, and toxicity prediction, therapy can be truly “tailor-made.” Finally, continued innovation in vector synthesis, gene editing, and automated manufacturing promises to reduce costs, moving this revolutionary approach from concept to a broadly accessible and sustainable routine care option.

In summary, the transition from “cell as a drug” to “body as a factory” represents not only a technological breakthrough but also a fundamental rethinking of therapeutic logic. Despite existing hurdles, through ongoing interdisciplinary integration and rigorous clinical iteration, in vivo CAR therapy is poised to overcome current barriers in the coming decade and deliver transformative solutions across a broad spectrum of diseases—including cancer, autoimmune disorders, and fibrotic conditions.

## Data Availability

The datasets used and/or analyzed during the current study are available from the corresponding author upon reasonable request.

## Abbreviations

AAV: Adeno-Associated Virus
Adapter-LV: Adapter-Lentiviral Vector
AU: Arbitrary Units
B-ALL: B-cell Acute Lymphoblastic Leukemia
BCMA: B-cell Maturation Antigen
CAIX: Carbonic Anhydrase IX
CAR: Chimeric Antigen Receptor
Cas9: CRISPR-associated protein 9
CD3-LV: CD3-targeted Lentiviral Vector
CD8-LV: CD8-targeted Lentiviral Vector
circRNA: Circular RNA
C-index: Concordance Index
CODEX: CO-Detection by indEXing
CRISPR: Clustered Regularly Interspaced Short Palindromic Repeats
CRP: C-reactive Protein
CRS: Cytokine Release Syndrome
CSR: Chimeric Signaling Receptor
ctDNA: Circulating Tumor DNA
CXCL9: C-X-C Motif Chemokine Ligand 9
CXCL10: C-X-C Motif Chemokine Ligand 10
CXCL11: C-X-C Motif Chemokine Ligand 11
CXCL12: C-X-C Motif Chemokine Ligand 12
CXCR3: C-X-C Motif Chemokine Receptor 3
CXCR4: C-X-C Motif Chemokine Receptor 4
DNAM-1: DNAX Accessory Molecule-1
ECM: Extracellular Matrix
ERBB2: Erythroblastic Oncogene B2
FAP: Fibroblast Activation Protein
FAS: Fas Cell Surface Death Receptor
FasL: Fas Ligand
GM-CSF: Granulocyte-Macrophage Colony-Stimulating Factor
GMP: Good Manufacturing Practice
HER2: Human Epidermal Growth Factor Receptor 2
HLA-E: Human Leukocyte Antigen E
HLA-I: Human Leukocyte Antigen I
H-score: Histochemical Score
IHC: Immunohistochemistry
ICANS: Immune Effector Cell-Associated Neurotoxicity Syndrome
IFN-γ: Interferon-gamma
IL-2: Interleukin 2
IL-7: Interleukin 7
IL-10: Interleukin 10
IL-15: Interleukin 15
IMC: Imaging Mass Cytometry
iNOS: Inducible Nitric Oxide Synthase
KIRs: Killer Cell Immunoglobulin-like Receptors
KLRC1: Killer Cell Lectin-like Receptor C1
LAG-3: Lymphocyte-Activation Gene 3
LNP: Lipid Nanoparticle
LV: Lentiviral Vector
MHC: Major Histocompatibility Complex
MICA/B: MHC Class I Polypeptide-Related Sequence A/B
MMP: Matrix Metalloproteinase
MOG: Myelin Oligodendrocyte Glycoprotein
mRNA: Messenger RNA
mRNA-LNP: MRNA Lipid Nanoparticle
mxIF: Multiplex Immunofluorescence
NAbs: Neutralizing Antibodies
NKG2A: Natural Killer Group 2A
NKG2D: Natural Killer Group 2D
PBAE: Poly(β-amino ester)
PD-1: Programmed Cell Death Protein 1
PD-L1: Programmed Death-Ligand 1
PSMA: Prostate-Specific Membrane Antigen
RES: Reticuloendothelial System
scFv: Single-Chain Variable Fragment
SIRPα: Signal-Regulatory Protein α
ST: Spatial Transcriptomics
Syk: Spleen Tyrosine Kinase
TAA: Tumor-Associated Antigen
TAMs: Tumor-Associated Macrophages
TCF1: T-Cell Factor 1
Tex: Terminally Exhausted T cells
Tpex: Precursors of Exhausted T cells
TGF-β: Transforming Growth Factor-beta
TIGIT: T Cell Immunoreceptor with Ig and ITIM Domains
TILs: Tumor-Infiltrating Lymphocytes
TIM-3: T Cell Immunoglobulin and Mucin Domain-3
TME: Tumor Microenvironment
TOX: Thymocyte Selection-Associated High Mobility Group Box
TRAIL: TNF-Related Apoptosis-Inducing Ligand
Treg: Regulatory T cells
TSA: Tumor-Specific Antigen
VEGF: Vascular Endothelial Growth Factor
